# Blockchain-enabled cross-chain collaboration model for elderly health information from a whole process perspective

**DOI:** 10.3389/fpubh.2023.1081539

**Published:** 2023-03-10

**Authors:** Mo Hu, Fan Sheng

**Affiliations:** ^1^School of Journalism and Communication, Nanjing Normal University, Nanjing, China; ^2^School of Economics and Management, Harbin Engineering University, Harbin, China

**Keywords:** blockchain, cross-chain collaboration, elderly, health information, virtual chain

## Abstract

Due to people having less children and the aging population, the demand for elderly health services is increasing, which leads to an increase in demand for elderly health information. However, there is a gap between elderly medical health information and elderly care information due to different storage institutions and storage methods, which makes it difficult for the medical service industry and the elderly service industry to fully grasp and utilize the health information of the elderly. Therefore, it is difficult to provide whole process services that combine elderly medical health and elderly care. To solve the problem of the poor collaborative utilization of elderly healthcare information, this paper, based on blockchain cross-chain technology and the literature and field research, studies the specific contexts that are needed to realize elderly health information collaboration. Based on the system theory viewpoint, the component-based modular design concept is used to identify the attributes and types of current health information of the elderly from health information related to the five modules of prevention, detection, diagnosis, treatment, and rehabilitation in the process of elderly healthcare. This paper explores the structure, elements, and interactions between the medical health information chains and the elderly care information chains. We build a blockchain-enabled cross-chain collaboration model of elderly health information from the perspective of the whole process with the help of the underlying logic of virtual chain, and to realize the applicability and flexibility of cross-chain collaboration for health information for the elderly in the whole process. The research results show that the proposed cross-chain collaboration model can realize the cross-chain collaboration of health information for the elderly with easy implementation, high throughput, and strong privacy protection.

## 1. Introduction

According to the United Nations Population Division, the world is currently facing a continuous change in the age structure of the population, and as life expectancy increases and the trend toward having fewer children continues, population aging is expected to increase globally, with the size of the elderly population expected to more than double by 2050 (1). This will lead to an increasing number of older adults requiring treatment for age-related diseases and care for daily living, resulting in an increasing demand for medical and elderly care services and related assistive technologies, which will lead to a trend for older adults to live in elderly care institutions (2). However, with the limited resources available from the government, society, and families, securing the quality of life for the elderly is in conflict with the rapid increase in the number of elderly people.

Although increasing the supply of resources in the field of elderly care can play a role in alleviating conflicts to a certain extent, resources cannot be invested indefinitely, which requires maximizing the efficiency of the utilization of limited resources. On the one hand, new technologies can be developed to expand the categories and quality of medical and nursing services and achieve qualitative improvement, but this requires time and resource accumulation. On the other hand, the current problem can be solved with minimum resource consumption through optimal allocation and efficient utilization (3). Moreover, alleviating conflicts through the latter can provide more space for technological progress, so when facing the above problems, effective solutions can be sought from currently available information technologies. Compared with the decentralized services, systems that integrate health care and social elderly services and achieve collaboration between them play an important role in solving health problems of the elderly (4, 5). The effective application of blockchain cross-chain technology to achieve cross-chain collaboration between elderly medical health information and elderly care information is one of the alternatives to effectively achieve this goal. In general, medical institutions hold the medical health information of the elderly, while the elderly care information is stored in different subjects according to the type of elderly care. For instance, the elderly care information in home care is usually in the hands of the elderly themselves, their family members or family doctors; the information in community care is usually in the hands of community caregivers; and the information in institutional pension is usually in the hands of the institutions they live in. At present, due to limited medical resources, for countries and regions that are less developed, few elderly people have access to the long-term services of family doctors or caregivers, and it is difficult to obtain comprehensive health information for the elderly through such ways. Moreover, the care expertise, skills and equipment possessed by most elderly people and their families are often make it difficult to support the accurate acquisition of elderly health information. Compared with home care and community care, formal elderly care institutions have more advantages in obtaining comprehensive elderly care health information consistently and accurately. Therefore, based on the current objective conditions of personnel, technology, and equipment, when exploring ways to achieve optimal allocation and efficient utilization of medical and elderly care resources through the collaboration of elderly medical health information and elderly care information, it is most likely to be realized at the practical level by starting from the collaboration of health information in medical institutions and elderly care institutions. Therefore, this study will focus on how medical institutions and elderly care institutions can provide whole process integrated elderly care and medical services through emerging information technology when facing the needs of the elderly.

To solve this problem, adequate collaboration between the two systems needs to be achieved, and the key challenge in this process is how to achieve the collaboration of information (6). The specialized and complex nature of health information makes the verification, storage, and sharing of health information challenging, while the decentralized, highly secure, and tamper-proof nature of blockchain technology, as well as suitability to IoT, make it naturally advantageous in the sharing of health information (7). In order to guarantee the efficiency of the system's operation, the results should be fully linked to its operation process, so that the efficient operation of the collaborative system can be achieved through good management of the process (8). In view of this, based on the whole process perspective, this paper will combine the research of Albahri et al. (9) and Walker (10) with the current situation of healthcare for the elderly, and comprehensively discuss the roles played by medical institutions and elderly care institutions from five aspects: prevention, detection, diagnosis, treatment, and rehabilitation, as well as how to realize the elderly health information collaboration based on blockchain technology between the two. Thus, it can be used to provide quality health information services for the elderly in the whole process, thereby laying the foundation for the optimal allocation and effective utilization of resources.

In this paper, based on a review of existing research, we discuss the composition of the whole process of healthcare for the elderly (i.e., prevention, detection, diagnosis, treatment, and rehabilitation) and the roles of medical institutions and elderly institutions in different modules of the whole process. Then, we explore how to achieve information collaboration and effective operation between medical institutions and elderly care institutions through blockchain technology in order to realize the efficient linking of the five modules.

## 2. Literature review

Health informatics aims to apply the power of information technology to improve the quality and efficiency of care for patients and specific populations (11). Currently, health informatics research focuses on health information literacy, health information behaviors, health information needs, and health information resource management. (i) In the field of health information literacy, Taheri et al. (12) pointed out that people's health-related decisions are closely linked to their health information literacy. Hassan and Masoud (13) studied the health information literacy of non-medical college students in terms of gender differences and showed that health information literacy was positively correlated with female gender and frequency of online health information searches. (ii) In a study of health information behavior, Lee and Oh (14) analyzed the health information retrieval behavior of people in the 40–50 age group, and the results showed that those with greater health consciousness and health information orientation considered official information on government websites as an important information cue when retrieving health information. An and Jeong (15) conducted a survey on a sample of 240 older adults aged 65 years or older in order to improve their understanding of older adults' online health information behaviors. The results of the study showed that health consciousness was positively associated with empowerment, and empowerment was positively correlated with information behaviors (15). (iii) In the study of health information needs, Gao (16) analyzed the complementary and alternative value of online health information based on data from the 2020 National Health Information Trends Survey in the U.S. The results of the study indicated that online health information can be an alternative source when it is difficult to use traditional health information resources. Jang (17) obtained and analyzed the health information usage of 171 immigrants through a questionnaire; the results showed that most immigrants do not actively use health information in general and that health information on the Internet is the main source of information for immigrants as they age and when illness occurs. (iv) In terms of health information resource management research, Strekalova (18) states that more than 90% of U.S. hospitals provide electronic medical records to patients, but the use of electronic medical records by patients remains low. Ifeoluwa (19) states that a well-structured and coordinated health information management system can generate the information needed to make decisions at all levels of healthcare services. Therefore, this paper focuses on health information collaboration, which falls under the research category of health information resource management and, at the same time, information collaboration.

The existing research on information collaboration mainly focuses on information collaboration network construction and information collaboration application practice. (i) In the research of information collaboration network construction, Yang (20) proposed a computer-aided measurement method for a multi-source information collaboration network structure of smart cities based on an adaptive artificial immune network algorithm for the problem of low measurement efficiency. Compared with a genetic algorithm, this method has faster convergence speed and higher prediction accuracy. Ezequiel et al. (21) proposed a framework for autonomous decentralized vehicle regulation based on virtual vehicle information collaboration, which can identify the geographic location of virtual vehicles, upload the willingness motivation information, and request cooperation information of different virtual vehicles back to the platform, thus realizing information collaboration among different virtual vehicles. (ii) In terms of practical research on the application of information collaboration, Jiang (22) points out that information collaboration has become a difficult aspect of supply chain management. He uses IoT and big data technologies to establish a simulation model of the supply chain based on the bullwhip effect, and the images of simulation results objectively clarify the value of information collaboration in the supply chain. Wang et al. (23) points out that the critical infrastructure system consists of multiple critical infrastructures, and different critical infrastructures are functionally and structurally interdependent. Thus, the adoption of information collaboration to achieve data transfer between these critical infrastructures is important for supporting the daily work of the critical infrastructure system. The purpose of this paper is to investigate elderly health information collaboration, which is a specific application of theories and methods related to information collaboration to the field of health information resource management research. Generally, medical health information and elderly care health information are stored in separate information chains. Thus, an important prerequisite to realize elderly health information collaboration, so as to provide the whole process of health services for the elderly, is to solve the cross-chain problem between the medical health information chain and the elderly care health information chain.

In recent years, the application of information technology in the field of health information management has received increasing attention from researchers (24). Among this research, the emerging cross-chain technology of blockchain is designed to mediate trust building and information transfer between different blockchains (25). This concept fits with the design concept of this study's cross-chain collaborative model of health information for the elderly. In view of this, this paper explores the possibility of employing cross-chain technology in blockchain to solve the cross-chain problem between the medical health information chains and the elderly care information chains. The cross-chain technology of blockchain is an important technology in realizing the interconnection and value transfer of information between different blockchains (26). Existing research has focused on the implementation mechanism of cross-chain technology of blockchain, such as Ye et al. (27), who designed a heterogeneous blockchain interoperability platform based on sidechain relay, which allows asset exchange, information interaction, and service complementation between heterogeneous blockchains. Monika et al. (28) proposed a method for atomic cross-chain transfer between heterogeneous blockchains with a view to data sharing and mutual interaction between different blockchains. Meanwhile, research on enhancing blockchain user experience by combining blockchain design with perceived user experience is becoming increasingly mature, and these studies provide the theoretical basis for the realization of this study (29, 30). This study will draw on the cross-chain technology and ideas of the above-mentioned research to explore the realization path and solve the problem of medical and elderly care information collaboration.

In summary, elderly health information collaboration belongs to the intersection of health information resource management and information collaboration. Currently, although there is a wealth of research in both areas, there is relatively little literature on health information collaboration research. Health information collaboration helps to aggregate and share health information about a patient or a specific population from multiple sources (31). Older adults are the largest consumers of healthcare and generate a lot of health information stored in different institutions (32), and it is necessary to study the issue of health information collaboration in the elderly population in order to effectively use these different sources of health information and provide better healthcare services to older adults. Meanwhile, emerging information technologies (e.g., cross-chain technology of blockchain) have been able to support the realization of cross-chain collaboration of health information for the elderly at the information technology level. In view of this, this paper adopts the cross-chain technology of blockchain to study the health information collaboration model between medical institutions and elderly care institutions based on the whole process perspective. Thus, the paper aims to provide more comprehensive health data support for medical decision makers, so as to provide more high-quality and efficient healthcare services for elderly people. At the same time, even for those countries and regions where medical services, elderly care services, and Internet infrastructure construction are underdeveloped, this study can provide references for them to establish elderly health information databases, improve the elderly medical health and elderly care system, and build elderly health information chain in the future. This may provide a theoretical basis for underdeveloped countries and regions to gradually build a whole-process cross-chain collaborative model for elderly health information construction.

## 3. Identification of health information chains for elderly in the context of the whole-process health service

### 3.1. Materials and methods

In this study, 12 triple-A hospitals (top level) and 18 model elderly care institutions in northeast, north, east, central, south, southwest, and northwest China were investigated respectively. (1) In terms of identifying whole process elderly health information modules, through in-depth interviews with medical and nursing staff common diseases such as cardiovascular, cerebrovascular and respiratory diseases, and combined with the results of literature research, five elderly health information modules were finally identified. (2) In the identification of elderly medical health information chain, the information was mainly obtained through the interviews with the staff of the data management sections and the retrieval of public information of the hospitals mentioned above, and thus the current situation, obstacles, breakthroughs and expected goals were found. (3) As for the identification of the elderly care information chain, the information was obtained through the interviews with the staff of the data management departments and the retrieval of public information of the elderly care institutions mentioned above, after analyzing the relevant information, the current situation, difficulties, breakthroughs and expected goals of elderly care information chain construction were found.

Since this study mainly focuses on the macroscopic exploration of the cross-chain collaboration model of elderly medical health information and elderly care information, and does not actively access and use a specific individual's health information, so it does not involve moral and ethical aspects.

### 3.2. Analysis of elderly health information components

Sockolow et al. (33) concluded that the health information of the elderly should be recorded and adequately coordinated among the relevant medical institutions to ensure that the relevant personnel can make accurate judgments about the health conditions of the elderly based on sufficient information. Therefore, to ensure the smooth operation of the system, health information should be used throughout the five modules of prevention, detection, diagnosis, treatment, and rehabilitation in the process of healthcare for the elderly. Prevention is about reducing the risk of diseases in the elderly through diet, exercise, education, etc. Detection is about detecting health problems and symptoms in the elderly. Diagnosis refers to making judgments about the diseases or conditions of the elderly based on their health information and reality. Treatment is about helping the elderly to fight against diseases and disorders through medical means. Finally, rehabilitation in this paper mainly refers to promoting the elderly's return to normal functioning of their body and mind by means of care, spiritual comfort, and nursing.

The current decline in physician-centered healthcare models and the growing development of shared decision-making models of care have greatly increased the importance of health information for both physicians and patients and have prompted older adults to seek more ways to manage and use health information *via* the Internet (34). Combining information from the Centers for Medicare and Medicaid Services' PACE program (Program of All-Inclusive Care for the Elderly) in the United States and information from Sockolow et al. (33), Kumar et al. (35), and Vuuren et al. (36), we found that the health information about the elderly involved here is multifaceted, including basic personal information (e.g., age, gender, ethnicity), information related to activity of daily living (ADL) (e.g., eating, washing, going to the toilet, dressing, turning, walking), information on vital signs (e.g., breathing, temperature, pulse, blood pressure), medical records (e.g., medication, consultation, medical history, medical advice), family situation, social situation, and other objective information. It also includes the subjective information generated during the process of healthcare, such as subjective feelings about the care received (e.g., complaints, concerns, discomfort) and opinions on the treatment (e.g., whether they are willing to accept a particular treatment, whether they can accept the side effects). The complexity of managing this vast amount of information requires blockchain technology to enable doctors, caregivers, and family members to respond in a timely manner by accessing, storing, and sharing information about the health of older adults in real time. Through blockchain applications, older adults' health information records are stored confidentially, and the healthcare providers they meet can scan this information through a legalized process and assign them to a dedicated doctor or caregiver. In turn, the health information about the elderly is transparent to the individuals involved in the healthcare chain, and they are able to track or update it based on their identity location at any time and from any place, guaranteeing the security and ease of use of health information (35).

### 3.3. Identification of health information chains for elderly

The health information about the elderly runs through the five modules mentioned above. The health information in different modules is stored in different institutions and platforms, gradually forming the medical health information chain and the elderly care information chain. The medical health information chain for the elderly stores information related to medical care in the five modules acquired by medical institutions in the process of providing medical services for the elderly. The elderly care information chain, on the other hand, stores the health information related to elderly care in the five modules collected by elderly care institutions through daily care for the elderly. The information related to both medical services and daily care for the elderly are crucial in the implementation of the whole process of health services for the elderly. In this paper, we identify the current situation of two information chains for the elderly through a combination of literature research and field research. Thus, later, based on the clarification of the current situation of the medical health information chain and the elderly care information chain for the elderly, the cross-chain collaboration of the health information chain for the elderly can be realized through information technology (blockchain).

#### 3.3.1. Identification of elderly medical health information chains

**Figure 1:**
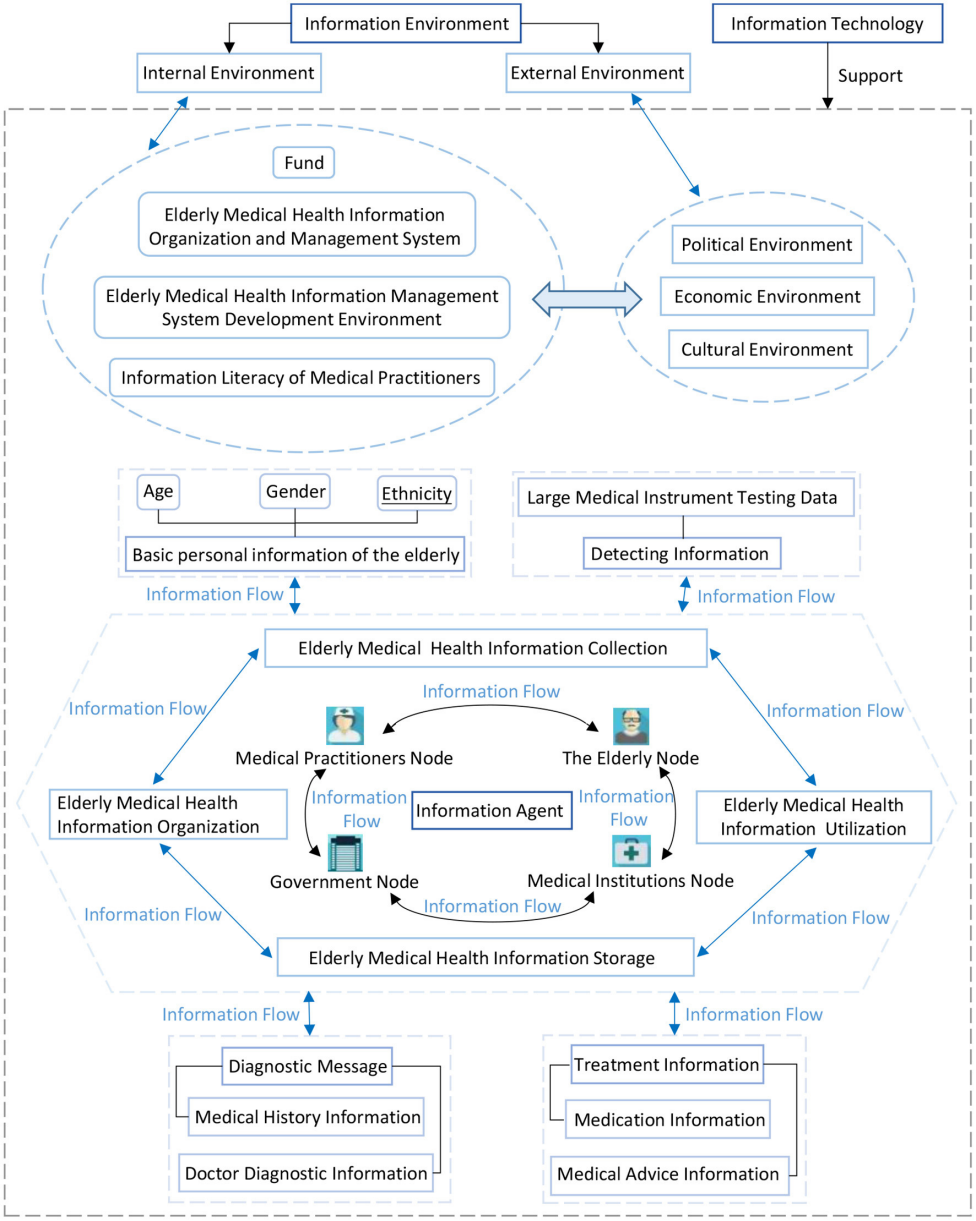
Components of the elderly medical health information chain and their interactions.

Through the previous literature review and field research and network research in many triple-A hospitals in China, the composition of the current medical health information chain for the elderly was analyzed. The medical health information chain of the elderly mainly consists of three parts: elderly medical health information, elderly medical health information subjects, and elderly medical health information environment. Among these, medical health information on the elderly mainly consists of basic personal information of the elderly, detection information (e.g., large medical instrument test data), diagnostic information (e.g., in terms of medical history information and doctor's diagnosis information), and treatment information (e.g., medication information and medical advice information). The medical health information subject includes relevant government departments that supervise the medical industry, medical institutions, medical personnel, and the elderly. The medical health information environment of the elderly can be divided into two categories: internal environment and external environment. Internal environment mainly consists of the organization and management system of medical health information for the elderly, the medical health information management system development environment for the elderly, financial support, information literacy of medical personnel, etc. The external environment mainly comprises the political environment, economic environment, and cultural environment in which the health information for the elderly is located. The components of the elderly medical health information chain and their relationships are shown in Figure 1.

As shown in Figure 1, the elderly medical health information chain consists of many components, which can be broadly divided into information environment, information people, and information according to their attributes, and the relationship between them is interdependent. In the information environment component, there are many sub-elements such as capital, the organization and management system of elderly medical health information, the elderly medical health information management system development environment, information literacy of medical practitioners, political environment, economic environment, and cultural environment. Among them, capital, the organization and management system of elderly medical health information, the elderly medical health information management system development environment, and the information literacy of medical practitioners are the micro-environment that affects the operation of the elderly medical health information chain. The political, economic, and cultural environments are the macro-environment that affects the operation of the elderly health information chain, which is called the external environment in this paper. The internal environment and the external environment influence and interact with each other, and together form the information environment in which the elderly health information chain exists. The information component mainly contains basic personal information, detection information, diagnosis information, and treatment information about the elderly. Different modules contain different sub-elements, for example, basic personal information of the elderly contains information such as age, gender, and race; detection information contains information such as test data of large medical instruments; diagnosis information contains the sub-elements of medical history information and doctor's diagnosis information, etc.; and treatment information contains medical history and medical advice information. The information subject elements mainly contain relevant government departments that supervise the medical industry, medical institutions, medical personnel, and the elderly, each of which can be regarded as a type of node in the medical health information chain of the elderly, and many different types of nodes are important components of the elderly medical health information chain. The complicated and heterogeneous information elements flow between different information subjects (i.e., nodes) in an orderly manner to create an information flow. Meanwhile, according to the flow of information elements between information people, information people elements can be divided as follows: elderly medical health information collection, elderly medical health information organization, elderly medical health information storage, and elderly medical health information utilization.

#### 3.3.2. Identification of elderly care information chains

**Figure 2:**
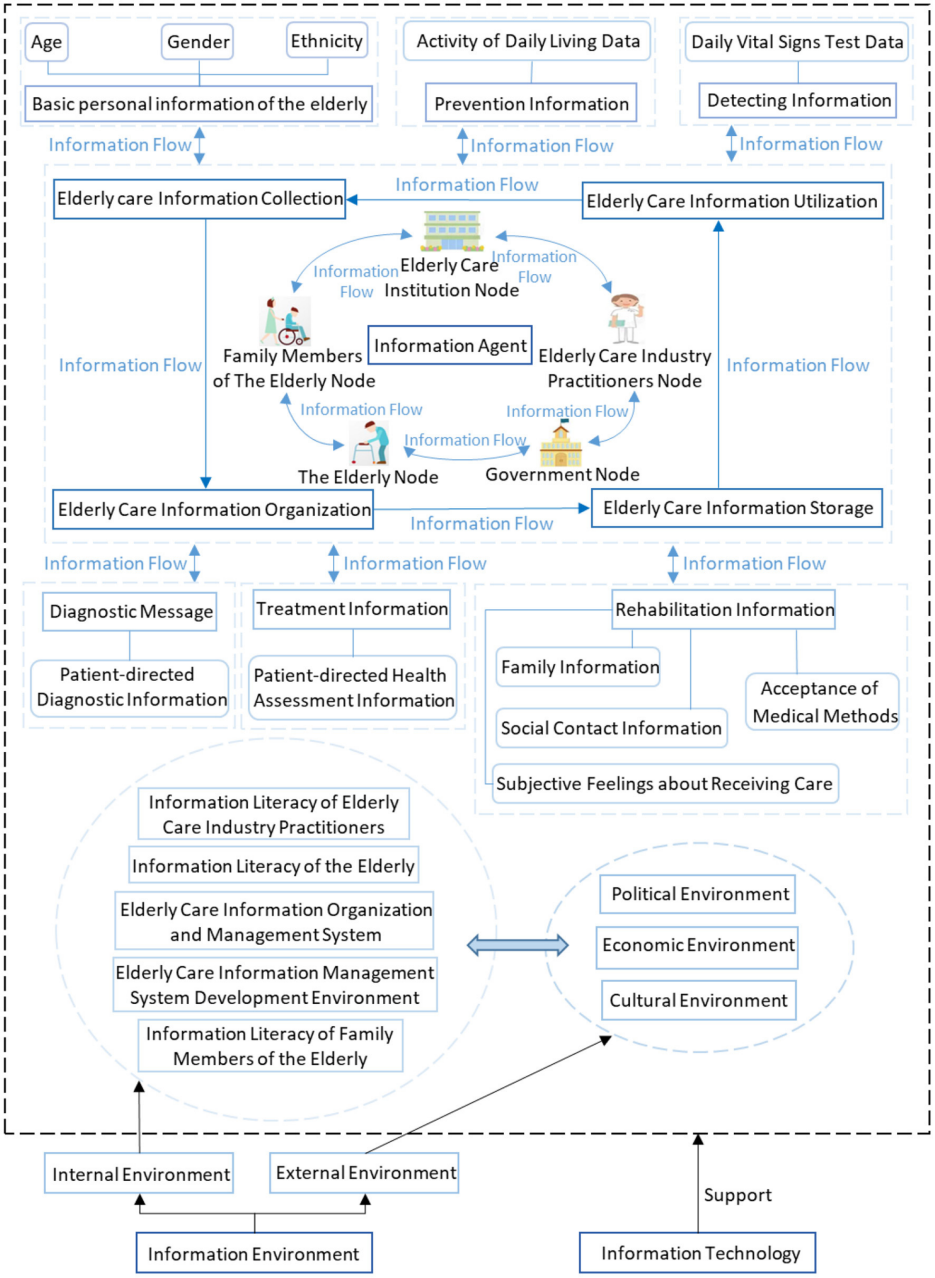
Components of elderly care information chain and their interactions.

The analysis of the elderly care information chain in this paper is based on the previous literature review and the authors' field research and network research on many model elderly care institutions in China. The current elderly care information chain mainly consists of three parts: elderly care information, elderly care information subjects, and elderly care information environment. Among them, the elderly care information includes basic personal information, prevention information, detection information (e.g., daily vital signs data monitoring), diagnosis information (e.g., patient-directed diagnostic information), treatment information (e.g., patient-directed health assessment information), and rehabilitation information. The elderly care information subjects include governmental supervisory departments of the elderly care industry, elderly care institutions, elderly people, family members of the elderly, and elderly care industry practitioners. The elderly care information environment consists of information literacy of elderly care industry practitioners, information literacy of the elderly and their family members, elderly care information organization and management system, elderly care information system development environment, political environment, economic environment, cultural environment, etc. Among them, political environment, economic environment, and cultural environment form the macro elderly care information environment, and the rest form the micro elderly care information environment. The components of the elderly care information chain and their interactions are shown in Figure 2.

As shown in Figure 2, according to the attribute, the elderly care information chain comprises three elements: information people, information, and information environment, and all elements influence and interact with each other to guarantee the operation of elderly care health information. The information people element contains five sub-elements: elderly care industry, elderly people, elderly people's families, government, and elderly care industry practitioners. Each sub-element can be regarded as a type of node, and numerous different types of nodes are important parts of the information chain for the elderly. Information flows between the different nodes of the elderly care information chain, and the information elements are equally important components of the elderly care information chain. The information elements include six parts of personal basic information, prevention information, detection information, diagnosis information, treatment information, and rehabilitation information about the elderly, each part contains different sub-elements. For example, personal basic information contains sub-elements such as age, gender, and ethnicity; prevention information contains sub-elements such as daily life activity data; detection information contains sub-elements such as daily vital signs data; diagnosis information contains sub-elements such as patient-directed diagnostic information; treatment information contains sub-elements of patient-directed health assessment information; rehabilitation information contains sub-elements such as family situation information, social situation information, subjective feelings about the care received, and suggestions for medical decisions. The flow of information in different modules among different nodes needs to go through four stages: elderly care information collection, elderly care information organization, elderly care information storage, and elderly care information utilization. The information environment elements of the elderly care information chain consist of internal environment (i.e., micro environment) and external environment (i.e., macro environment). The micro environment includes the information literacy of elderly care industry practitioners, the information literacy of the elderly and their family members, the elderly care information organization and management system, and the elderly care information system development environment. The external environment includes political environment, economic environment, and cultural environment. The interaction between the sub-elements of external environment and internal environment forms the information environment in which the elderly care information chain is located, and influences the flow of information elements and information behavior of information people elements in the elderly care information chain.

## 4. Blockchain-enabled whole-process elderly health information cross-chain collaboration model construction

### 4.1. Blockchain technology enabling cross-chain collaboration of health information for the elderly

As mentioned above, the health information of the elderly is stored in elderly medical health information chains and elderly care information chains separately, and this independence has led to the increasingly prominent problem of silos of elderly health information. Faced with the mass multi-source heterogeneous health information of the elderly, how to integrate this information, realize the cross-chain collaboration of whole process elderly health information, and then provide better health services for the elderly is a realistic problem that needs to be solved. As blockchain technology enters the 3.0 era, blockchain 3.0 technology, with cross-chain technology as the main breakthrough direction, solves the problem of information transmission between different blockchains, thus providing information technology support for realizing cross-chain collaboration of elderly health information in the whole process.

This study adopts the underlying logic of a virtual chain for cross-blockchain transactions proposed by Wang et al. (37), combines it with the specific application context of the collaboration between elderly medical health information chains and elderly care information chains, and builds a blockchain-enabled cross-chain collaboration model for health information for the elderly from a whole-process perspective based on a component-based modular design concept according to a systems theory perspective. The main reasons for choosing the underlying logic of their proposed virtual chain to solve the problem of cross-chain collaboration between elderly medical health information chain and elderly care information chain in a whole-process perspective are as follows. (i) Their proposed virtual chain has been proved to solve the problem of interoperability between different blockchains, and the cross-chain collaboration model of elderly health information to be built in this paper was precisely designed to solve the problem of interoperability between the elderly medical health information chain and the elderly care information chain. (ii) The virtual chain proved to be capable of guaranteeing the tamper-proof and traceable cross-chain transactions between different blockchains, and also controlling the access rights. One of the objectives of the cross-chain collaboration model of elderly health information is to realize the cross-chain collaboration of elderly health information through the whole process on the basis of guaranteeing the authenticity of all the elderly health information participating in the cross-chain collaboration and protecting the health privacy of the elderly. (iii) The virtual chain uses data fusion technology to achieve a significant increase in the throughput of cross-chain transactions. The amount of health information about the elderly is large and heterogeneous from multiple sources. In particular, in the case of medical image information, where the information that needs to be stored and interacted with is even larger, how can such a large amount of information be collaborated across chains? In summary, the underlying logic of the virtual chain has a good match with the functions expected to be achieved by the cross-chain collaboration model of elderly health information from the whole process. Based on the underlying logic of the virtual chain proposed by Wang et al. (37) and the specific context of the cross-chain collaboration of elderly health information, we built a blockchain-enabled cross-chain collaboration model of elderly health information from a whole process perspective.

### 4.2. Building a cross-chain collaboration model based on component-based modular design for whole-process health information for the elderly

The elderly are the part of the population that most requires healthcare, and a huge amount of health information is generated in the process of providing healthcare. This elderly health information is stored in elderly medical health information chains and elderly care information chains separately to ensure that both sets of data can efficiently serve the whole process of elderly healthcare. Based on the underlying logic of the virtual chain constructed by Wang et al. (37), this study adopts a component-based modular design concept based on a systems theory perspective, and combines the specific context of cross-chain collaboration between medical health information chains and elderly care information chains to construct a cross-chain collaboration model for elderly health information in the whole process of health services.

**Figure 3:**
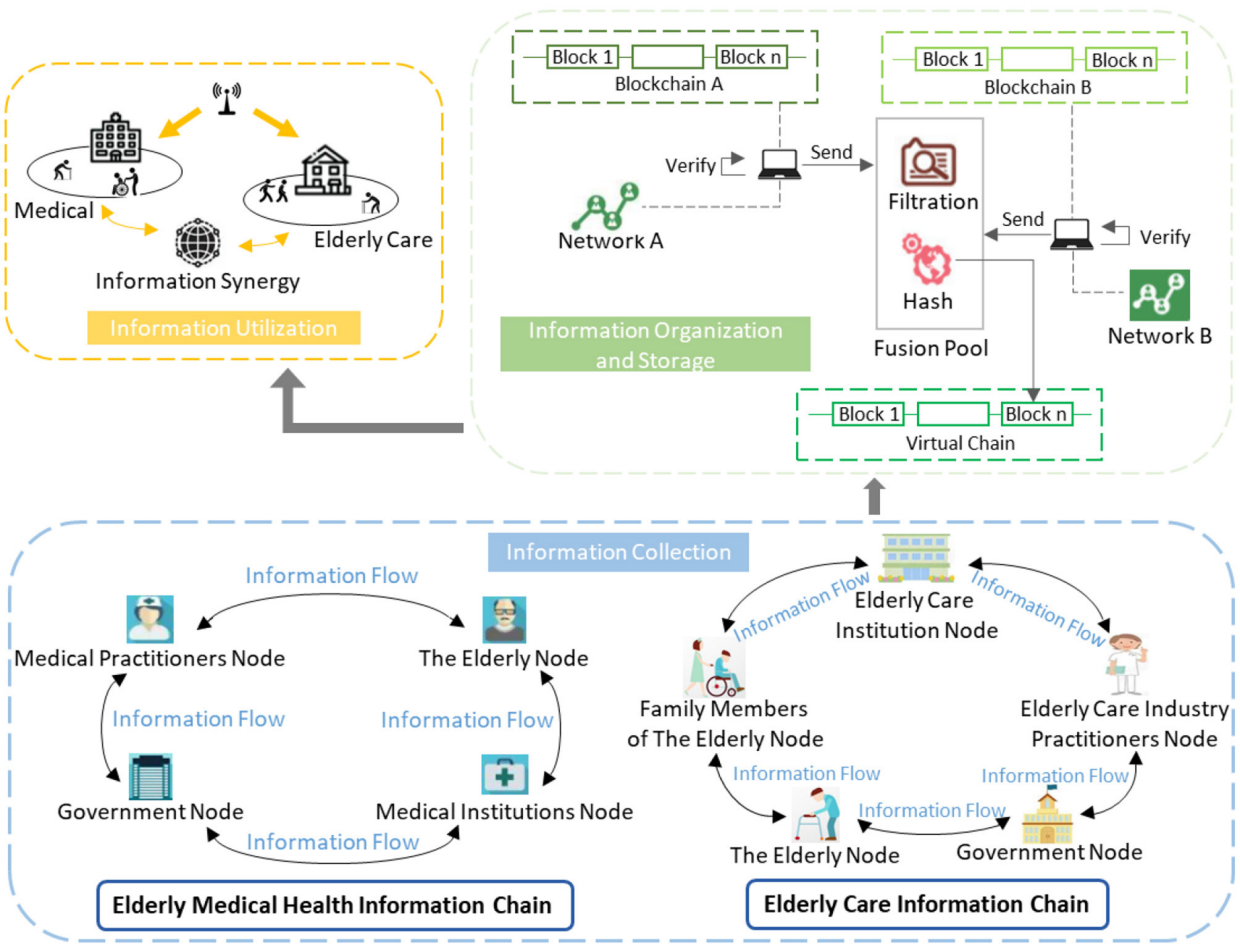
Blockchain-enabled whole-process elderly health information cross-chain collaboration model.

As shown in Figure 3, we constructed a blockchain-enabled cross-chain collaboration model (hereinafter referred to as the collaboration model) for the whole process of health information for the elderly according to the information flow. In the collaboration model constructed in this study, the process of information elements preserved in elderly medical health information chains and elderly care information chains flowing through the information people elements (i.e., nodes) are information collection, information organization, information storage, and information utilization. In the information collection stage, the collaboration model collected information from many different elderly medical health information chains and elderly care information chains according to the demand of the whole process of elderly health information collaboration. The information organization and information storage of the collaboration model constructed in this paper were realized by the virtual chain proposed and verified by Wang et al. (37). The implementation logic was as follows: the initiator of the cross-chain collaboration of elderly health information is set as the source chain and the receiver is the target chain. Both the source chain and the target chain provide health information of the elderly to the fusion pool and store it in the form of chains. The function of the fusion pool is to shield the underlying structure of different blockchains, thus realizing the cross-chain collaboration of multi-source heterogeneous information between different blockchains. The fusion pool only stores the block headers related to the cross-chain, which is designed to guarantee its information throughput in the process of cross-chain collaboration. The signature of the source and target chains is stored in the virtual chain to solve the problem of inconsistent distribution of different blockchain ledgers. In the cross-chain collaboration of elderly health information, the single information flow direction is one-way irreversible, which is used to prevent malicious tampering of information and to control access to health information of the elderly in different nodes. The above stage was followed by the information utilization stage, in which the elderly medical health information and the elderly health information from multi-source heterogeneous elderly people should be applied to the healthcare process. The ultimate goal of this paper is to find a way of ensuring the effective operation of the cross-chain collaboration of the whole process.

## 5. Conclusions

This paper aimed to explore the application of “blockchain+” in the field of healthcare for the elderly, and to promote blockchain underlying technology to serve the cross-chain collaboration of elderly health information from a whole-process perspective, which is an attempt to combine the future-oriented blockchain underlying technology to serve people's livelihood in a specific application context. Based on a literature review and field research, we found that the integration and utilization of health information for the elderly in China is inefficient, and the collaborative health information service across the whole process of elderly healthcare needs to be improved. To solve this problem, we firstly combined the results of the literature review and field research in medical institutions, elderly care institutions, and relevant government departments, and analyzed the five modules of prevention, detection, diagnosis, treatment, and rehabilitation. We further analyzed the specific types of elderly health information contained in each module. Then, we identified the structure, elements, and interactions among the elements of the elderly medical health information chain and elderly health information chain. On this basis, to realize the whole-process cross-chain collaboration of elderly health information, we constructed a cross-chain collaboration model of elderly health information from the whole-process perspective based on the virtual chain technology proposed and validated by Wang et al. (37), adopting a component-based modular design concept based on a systems theory perspective, combined with the specific context of cross-chain collaboration between medical health information chains and elderly care information chains.

The model has the following advantages: (i) Closeness to real needs. In a real-life situation, the health information of the elderly is distributed across various departments of medical and elderly care institutions, which often do not have the same storage structure for this information. The difficulty posed by this scenario is that the environment of the source chain is different from that of the target chain, and the data structures of the source and target chains are different. Without changing the current blockchain application infrastructure, the collaboration model constructed in this paper used the underlying logic of virtual chains to overcome this difficulty, which can achieve cross-chain collaboration between multiple elderly medical health information chains and elderly care information chains with different storage structures for elderly health information. (ii) Adopting the concept of component-based modular design makes the realization of the cross-chain collaboration model of elderly health information more convenient in terms of the whole process. Each different elderly medical health information chain and the elderly care information chain can be regarded as a module of the cross-chain collaboration model of elderly health information from a whole-process perspective. With the cross-chain technology, the cross-chain collaboration of health information for the elderly can be realized between different modules. At the same time, each of the modules can be added or deleted depending on the specific needs, thus increasing the flexibility and applicability of the cross-chain collaboration model of elderly health information from a whole-process perspective. (iii) Collaboration of the whole process of elderly health information based on the premise that their health privacy is protected. To protect the health privacy of the elderly during information collaboration, the collaboration model constructed in this paper adopted elliptic curve signature verification and trustworthiness access control to guarantee the privacy protection of elderly health information. (iv) High throughput of cross-chain information collaboration. The whole process of elderly health information collaboration involves a huge amount of health information of the elderly, especially when it comes to medical image information. The difficulty posed by this situation in the construction of the collaboration model is that the amount of information collaboration per second in cross-chain information collaboration is mainly bound by the limited block capacity. To solve this problem, we chose the virtual chain cross-chain technology, which uses data fusion technology to expand the capacity of blocks, thus achieving high throughput of blockchain-enabled cross-chain collaboration of elderly health information from a whole-process perspective.

There is still room for progress in our research. Future research could further collect multi-source heterogeneous health information about the elderly in different institutions to form a database. It could also verify and enhance the security and throughput of the blockchain-enabled cross-chain collaboration model of elderly health information constructed in this paper in the experimental development environment through simulation. On the other hand, we can further focus on the model, path and method of cross-chain collaboration between the elderly care information involved in home and community care and the elderly medical health information of medical institutions. The results obtained can be integrated into the whole process elderly health information cross-chain collaboration model built in this study in a component-based modular way, thus to make the model more complete and universal.

## Data availability statement

The datasets presented in this study can be found in online repositories. The names of the repository/repositories and accession number(s) can be found in the article/supplementary material.

## Author contributions

MH: writing and revising. FS: writing and providing revised advice. All authors contributed to the article and approved the submitted version.
